# Role of Oxidative Stress and the Identification of Biomarkers Associated With Thyroid Dysfunction in Schizophrenics

**DOI:** 10.3389/fphar.2021.646287

**Published:** 2021-04-29

**Authors:** Mahmood Rasool, Arif Malik, Shamaila Saleem, Muhammad Abdul Basit Ashraf, Altaf Qadir Khan, Sulayman Waquar, Ayesha Zahid, Sumaira Shaheen, Muhammad Abu-Elmagd, Kalamegam Gauthaman, Peter Natesan Pushparaj

**Affiliations:** ^1^ Department of Medical Laboratory Technology, Faculty of Applied Medical Sciences, King Abdulaziz University, Jeddah, Saudi Arabia; ^2^ Center of Excellence in Genomic Medicine Research, King Abdulaziz University, Jeddah, Saudi Arabia; ^3^ Institute of Molecular Biology and Biotechnology (IMBB), The University of Lahore, Lahore, Pakistan; ^4^ University College of Medicine and Dentistry, The University of Lahore, Lahore, Pakistan; ^5^ Department of Psychiatry, Ameer-Ud-Din Medical College, Lahore, Lahore, Pakistan; ^6^ Center for Research in Molecular Medicine, The University of Lahore, Lahore, Pakistan

**Keywords:** schizophrenia, hyperhomocysteinemia, oxidative stress, autoimmune thyroid diseases, biomarkers, antioxidants, reactive oxygen species, prolactin

## Abstract

**Background:** Schizophrenia is associated with a deficiency of dietary antioxidants like vitamin B6, B9, and B12 resulting in defective methylation leading to hyperhomocysteinemia. Hyperhomocysteinemia causes mitochondrial DNA damage, oxidative stress, vascular damage, and lipid peroxidation. Oxidative stress and increase in reactive oxygen species result in 8-oxodG production which induces apoptosis of both astrocytes and thyrocytes thus predisposing them to thyroid dysfunction and neurodegeneration. Furthermore, the presence of excessive free radicals increases thyroid thermogenesis causing hyperthyroidism or its excess may cause hypothyroidism by inhibiting iodide uptake. In the present study, we evaluated the various biomarkers associated with thyroid dysfunction in schizophrenics.

**Materials and Methods: **288 patients suffering from schizophrenia and 100 control subjects were screened for liver function tests (LFTs) such as alanine aminotransferase (ALT), aspartate aminotransferase (AST), alkaline phosphatase (ALP), and total bilirubin (TB). Also, the stress markers, namely malondialdehyde (MDA), homocysteine, cysteine, methionine, the thyroid profile including triiodothyronine (T3), thyroxine (T4), thyroid-stimulating hormone (TSH), thyroxine peroxide antibody (TPO-Ab); TSH receptor-Ab (TSHr-Ab), dietary antioxidants, lipids, cytokines, aminoacids and hormones, vitamins and trace elements, and other biochemical parameters.

**Results: **The LFTs showed elevated levels of ALT (45.57 ± 4.87 Vs. 26.41 ± 3.76 U/L), AST (40.55 ± 1.34 Vs. 21.92 ± 3.65 U/L), ALP (121.54 ± 4.87 Vs. 83.76 ± 5.87 U/L), and total bilirubin (2.63 ± 0.987 Vs. 1.10 ± 0.056 mg/dl), in schizophrenics than controls. Increased levels of MDA (3.71 ± 0.967 Vs. 1.68 ± 0.099) and homocysteine (17.56 ± 2.612 Vs. 6.96 ± 1.987 μmol/L were observed in schizophrenics compared to the controls, indicating increased stress. Levels of cysteine and methionine were decreased in schizophrenics than the controls (1.08 ± 0.089 Vs. 4.87 ± .924 μmol/L and 17.87 ± 1.23 Vs. 99.20 ± 5.36 μmol/L). The levels of TPO-Ab (IU/ml), Tg-Ab (pmol/L), and TSHr-Ab (IU/L) were observed to be higher in the patients’ group as compared to control subjects (9.84 ± 2.56 Vs. 5.81 ± 1.98, 55.50 ± 2.98 Vs. 32.95 ± 2.87 and 2.95 ± 0.0045 Vs. 1.44 ± 0.0023 respectively). Levels of Vitamin B6, B9, and B12 were also significantly decreased in the patients compared to the healthy controls.

**Conclusion: **The schizophrenics, demonstrated altered liver function, increased stress markers, and decreased dietary antioxidants. Reduced primary and secondary antioxidant levels, may result in hyperhomocysteinemia and cause further DNA and mitochondrial damage. Therefore, homocysteine and/or prolactin levels may serve as candidate prognostic markers for schizophrenia. Also, both neurological symptoms and the susceptibility to thyroid disorders may be prevented in the initial stages of this debilitating disorder by appropriate dietary supplementation of antioxidants which can rectify a reduction in primary and secondary antioxidants, and disturbed prolactin-serotonin-dopamine interactions in schizophrenics.

## Introduction

Schizophrenia is a neuropsychiatric disorder manifested by disruptive thinking, quantifiable language disturbances, perception, and self-sense (Barnham et al., 2004; Ciobica et al., 2011). It is characterized by disturbances in behavior, thinking ability, and gross distortion from reality (Ciobica et al., 2011). Among the psychiatric disorders, schizophrenia accounts for 1.1% of adults with nearly 21 million individuals being affected in the United States alone (Santos et al., 2012). It is among the seven most disabling diseases in patients between 20–45 years of age being more common than cardiovascular diseases, HIV, and diabetes. The identified causative agents for schizophrenia are stress, malnutrition, genetics, drugs, and alcohol abuse (Svrakic et al., 2013). The symptoms of schizophrenia are categorized into three groups: general (depression and hostility), positive (delusions and hallucinations), and negative (anhedonia and violation) (Barnham et al., 2004; Tamminga and Holcomb, 2005).

Schizophrenics have susceptibility toward thyroid disorders, and the reduction in catalase (CAT) levels results in elevated hydrogen peroxide (H_2_O_2_) thereby playing a potential role in thyroid hormogenesis (Adam-Vizi and Chinopoulos, 2006). Oxidative stress occurs in both astrocytes and thyrocytes as there is an increase in pro-oxidants and a decrease in antioxidants due to an increase in the production or decrease in the processing of reactive oxygen species (ROS) (Li et al., 2006). In schizophrenia, there is either reduction in primary antioxidants levels including superoxide dismutase (SOD), CAT and glutathione peroxidase (GPx), or a decrease in the secondary antioxidants such as glutathione, vitamins B6 (pyridoxine), B9 (folate), B12 (cobalamin), C, D, and E (Schweizer et al., 2008; Mitchell et al., 2014). The deficiency in vitamins B6, B9_**,**_ and B12 leads to enhanced homocysteine levels resulting in decreased glutathione and vitamins C and E levels mediating oxidative insult leading to neurotoxic effects (Lindqvist et al., 2017; Mikkelsen et al., 2017). Reduced vitamin C levels induce serotonin reduction and dopamine induction but on the other hand, levels of trace elements including copper and zinc have opposite effects resulting in hyperprolactinemia mediated depression (Pohl et al., 2011). Vitamin E has thyroid hormone suppressive action thus its deficient levels induce hyperthyroidism (Subudhi and Chainy, 2010).

The disturbed serotonin-dopamine-prolactin interactions are one side of the picture while the other aspect is deranged thyroid functions including higher thyroid-stimulating hormone (TSH) levels indicating hypothyroidism (Petrulea et al., 2012). It has been shown that dietary lack of L-arginine and an increase in asymmetrical dimethylarginine (ADMA) due to hyperhomocysteinemia reduces nitric oxide synthase (NOS) dependent nitric oxide (NO) production leading to schizophrenic symptoms. Glutamate is an important amino acid in the production of both glutathione and NO, and its deficiency mediates schizophrenic symptoms (Koga et al., 2011). Phenylalanine and tyrosine ultimately produce dopamine, but their reduced levels also induce an imbalanced serotonin-dopamine-prolactin relationship (Liao and Ya-Mei, 2014). In schizophrenics, there may be dietary insufficiency or poor exposure to sunlight resulting in vitamin D deficiency, which makes them susceptible to autoimmune thyroid disorders and is reflected by higher thyroid antibody levels (Kivity et al., 2011). The present study aimed to 1) determine the levels of primary and secondary antioxidants in the serum of schizophrenics, 2) assess homocysteine levels and their relation to thyroid dysfunction, 3) examine serotonin-dopamine-prolactin interactions and hyperprolactinemia mediated depression (HMD), 4) observe the schizophrenics susceptibility to thyroid disorders by measuring their thyroid profile and 5) elucidate vitamin D levels and their effects in rendering the schizophrenics to autoimmune thyroid disorders (AITDs).

## Materials and Methods

### Subjects

Two hundred and eighty-eight newly diagnosed patients (males and females) suffering from schizophrenia in the age group of 13–73 years were considered and included in the prospective study at the Department of Psychiatry, Social Security Hospital Lahore, Fountain House Lahore, and Mental Hospital Lahore, Lahore, Pakistan. One hundred sex and age-matched healthy individuals free from schizophrenia with an age range between 15 and 70 years were considered as controls. The study was conducted according to the ethical committee approval of the Institute of Molecular Biology and Biotechnology (IMBB), The University of Lahore (UOL) (IRB/IMBB/UOL/ph-984). Before the start of the study, written informed consent was obtained from all participants of the study according to Helsinki’s declaration.

### Inclusion and Exclusion Criteria

Newly diagnosed schizophrenia patients were included and any subject with a history of smoking, alcohol intake, or on antiparkinsonian/antipsychotic medications was excluded from the study (Duval et al., 2010; Bredin et al., 2013). All control subjects were healthy with no history of chronic diseases such as diabetes mellitus, liver diseases, and cancer (Duval et al., 2010; Duval et al., 2020).

### Blood Samples

Blood samples were collected from the cubital vein of each subject in appropriate tubes (with or without anticoagulant) for separation of plasma and serum separately. Plasma was separated immediately and for the serum separation, the blood samples were allowed to clot by leaving the sample at room temperature for 30 min. The clotted blood sample was then centrifuged at 3,000 rpm for 10 min and the clear supernatant was collected. Both the plasma and serum were aliquoted and stored at -80°C until analysis.

### Biochemical Analysis

All patients were assessed for their demographic profile and screened for their fasting blood glucose (FBG), gamma-glutamyl transferase (GGT), albumin (ALB), blood urea nitrogen (BUN), bicarbonate (HCO_3_), uric acid, and bilirubin using a semi-automated clinical chemistry analyzer as described before (Kumari et al., 2020).

### Estimation of Lipid Profile

The lipid profiles including total cholesterol (TCh), triglycerides (TGL), high-density lipoproteins (HDL), low-density lipoproteins (LDL) were estimated using commercially available kits (Sigma-Aldrich, United States).

### Liver Function Tests

Assays for liver function tests (LFTs) were performed including aspartate aminotransferase (AST), alanine aminotransferase (ALT), alkaline phosphatase (ALP), and total bilirubin (TB) using commercially available kits (Sigma-Aldrich, United States).

### Estimation of Antioxidant Profile

Glutathione was estimated based on the method described Moron et al., 1979. The 100 μl of plasma was taken and added 0.02 M (2.4 μl) EDTA and ice-cooled (10 min), Followed by the addition of 2 ml distilled water. To this 50.0 μl of TCA (50%) was added and incubated on ice for 15 min. Samples were then centrifuged (3,500 rpm). The supernatant was removed and added with 2 ml of 0.15 M Tris. HCl and 0.05 ml (DTNB). Absorbance was measured at 412 nm. Catalase (CAT) was determined by the method of Aebi, 1974. 100 μl of the sample was added to the tube followed by 1.9 ml of phosphate buffer and 1 ml of H_2_O_2_. The absorbance was measured after every minute at 240 nm. Superoxide dismutase (SOD) was estimated as per Kakkar et al., 1984. The 100 μl of the sample was taken in the tube, added with 1.2 ml of PBS, 100 μl of phenazine methosulfate, 300 μl of NBT, and 200 μl of NADH. Thereafter, 100 μl of Glacial acetic acid and 4 ml of 2-propanol were added further in the tube and centrifuged at 3,000 rpm for 10 min. The absorbance was taken at 560 nm. Glutathione peroxidase (GPX) was analyzed by Goldberg and Spooner, 1983, and Glutathione Reductase (GR) using commercially available kits (Sigma-Aldrich, United States). Malondialdehyde (MDA) was estimated using the method described by Ohkawa et al., 1979. 200 μl of the sample was taken in the tube, to which 200 μl of 8.1% SDS and 1.5 ml of 20% acetic acid were added. Later 1.5 ml of 0.8% TBA and 600 μl of distilled water along with 4 ml of 2-propanol was supplemented. It was centrifuged (4,000 rpm, 10 min) and the supernatant was removed for measuring absorbance at 532 nm using a UV-1100 spectrophotometer. Advanced Oxidative Protein Products (AOPPS) was estimated by the method of Witko-Sarsat et al., 1998. 200 μl of the sample was first diluted with PBS, then 10 μl of KI (1.16 M), and 20 μl of acetic acid was added. The sample was centrifuged at 5,000 rpm for 5 min and absorbance was measured at 340 nm on UV-spectrophotometer. Advanced glycation end products (AGEs) were estimated based on the method provided by Kalousova et al., 2002.

Nitric Oxide (NO) was estimated based on the method described before (Rasool et al., 2016). 100 μl of Griess Reagent was added with 300 μl of sample and supplemented with 2.6 ml of distilled water followed by incubation (30 min). The absorbance was measured at 548 nm. On the other hand, the myeloperoxidase (MPO), nitric oxide synthase (NOS), C - reactive protein (CRP), and ferritin levels were estimated using a commercial kit from Sigma-Aldrich, United States.

### Thyroid Profile

The thyroid profile [triiodothyronine (T3), thyroxine (T4), thyroid-stimulating hormone (TSH), thyroid-stimulating hormone receptor antibodies (TSHr-Ab), thyroid peroxidase (TPO), thyroglobulin (TG), levels of reverse triiodothyronine (rT3), levels were also measured using commercially available kits (Sigma Diagnostics, United States).

### Estimation of Vitamins and Trace Elements

Vitamin C was estimated based on the method described by Chinoy et al.,1986. 100 μl of the sample was added with 400 μl of TCA 5% and centrifuged at 3,000 rpm for 10 min. 320 μl of supernatant was separated and added with 130 μl of DTC and allowed to heat at 90° for 1 h. It was later ice-cooled and added with 600 μl of sulphuric acid then subjected to absorbance at 520 nm (Rasool et al., 2017). Vitamin E was estimated by the method of Rosenberg, 1992. 200 μl of the sample was supplemented with 200 μl of ethanol, 200 μl of n-hexane, and distilled water. It was then centrifuged at 3,000 rpm for 10 min and added with 25 μl of Bathophenanlhroline, 75 μl of ferric chloride, and 50 μl of Orthophosphoric Acid. Its absorbance was taken at 536 nm (Rasool et al., 2017). Both Vitamin A and Vitamin D were measured based on the methods described by Rutkowski and Grzegorczyk, 2007 and Arneson and Arneson, 2013 respectively. The vitamins (B6, B9, and B12) were estimated using the methods described before (Shaik and Gan, 2013). Sodium (Na) and potassium (K) were estimated by taking their absorbance with the help of a flame photometer (Kumar and Gill, 2018) and other trace elements in plasma were estimated based on the methods described previously (Bolann et al., 2007).

### Estimation of IL-2, IL-6, and TNF-alpha

Cytokines such as interleukins (IL-2, IL-6), and tumor necrosis factor-alpha (TNF-α) were analyzed using respective commercial enzyme-linked immunosorbent assay (ELISA) kits (BioVendor, Czech Republic).

### Aminoacids and Hormonal Profiles

The plasma aminoacids were estimated using the methods described by Shimbo et al., 2010. The levels of dopamine, serotonin, and prolactin were estimated using commercially available kits (Enzo Life Sciences Inc., United States).

### Statistical Analysis

All the statistical analysis was performed using SPSS version 21(IBM SPSS, United States). Variables were assessed with One-way ANOVA (*p* < 0.05) and Pearson Correlation was plotted.

## Results

The results from age and sex-matched controls and schizophrenics, in general, represent the physiological and biochemical parameters indicating the susceptibility and development of thyroid disorders in schizophrenics. The controls were selected to match the demographic profile of age, weight, body mass index (BMI), gender distribution, systolic, and diastolic blood pressure (Table 1).

**TABLE 1:** Demographic/physical characteristics.

Characteristics	Schizophrenics (*n* = 288)	Control (*n* = 100)
Age (Yrs)	13–73	15–70
Male (n)	162	47
Females (n)	126	53
Weight (kg)	21-77	20-80
SBP (mmHg)	133.48	130.21
DBP (mmHg)	84.59	81.24
BMI (kg/m^2^)	21.87	20.76

### Lipid Profile

The lipid profile of the schizophrenic patients was compared with the same number of controls (Table 2). The value of total cholesterol (Tch) was higher in schizophrenics (5.10 ± 1.76 mg/dl) than the control (4.49 ± 0.978 mg/dl). The LDL levels was also higher in schizophrenics (2.91 ± 0.564 mg/dl) than the control (2.31 ± 0.876 mg/dl). However, both the increases in Tch and LDL were statistically not significant. The levels of HDL was decreased in schizophrenics (1.31 ± 0.654 mg/dl) compared to the control (1.67 ± 0.265 mg/dl). Triglyceride levels was increased in schizophrenics (2.49 ± 0.056 mg/dl) compared to healthy control (1.40 ± 0.097 mg/dl). Both the decrease in HDL (*p* = 0.044) and the increase in triglycerides (*p* = 0.037) were statistically significant.

**TABLE 2:** Lipid profiles of schizophrenics versus controls.

Variables	Schizophrenics vs. control (mean ± SD)				*p*-values (**< 0.05)**
	Control	Schizophrenics	Schizophrenics (male)	Schizophrenics (female)	
TCh (mg/dl)	4.49 ± 0.978	5.10 ± 1.76	5.27 ± 1.76	4.97 ± 1.74	0.066
LDL (mg/dl)	2.31 ± 0.876	2.91 ± 0.564	2.89 ± 0.331	2.92 ± 0.86	0.097
HDL (mg/dl)	1.67 ± 0.265	1.31 ± 0.654	1.33 ± 0.422	1.30 ± 0.564	0.044
Tg (mg/dl)	1.40 ± 0.097	2.49 ± 0.056	2.50 ± 0.076	2.48 ± 0.069	0.037

### Liver Profile

Higher levels of ALT (45.57 ± 4.87 Vs. 26.41 ± 3.76 U/L), AST (40.55 ± 1.34 Vs. 21.92 ± 3.65 U/L), ALP (121.54 ± 4.87 Vs. 83.76 ± 5.87 U/L) and total bilirubin (2.63 ± 0.987 Vs. 1.10 ± 0.056 mg/dl) were found in schizophrenics in comparison to the corresponding controls (Table 3) and these increases in values were highly significant (*p* = 0.010, 0.014, 0.018 and 0.034 respectively). The levels of total protein (6.57 ± 1.56 mg/dl vs. 6.46 ± 1.65 mg/dl) in schizophrenics and controls respectively did not show much difference. However, serum albumin was significantly (*p* = 0.045) reduced in schizophrenics (2.71 ± 0.765 mg/dl) compared with healthy controls (2.79 ± 0.456 mg/dl).

**TABLE 3:** Hepatic profiles of schizophrenics versus controls.

Variables	Schizophrenics vs. control (mean ± SD)				*p*-values (**< 0.05)**
	Control	Schizophrenics	Schizophrenics (male)	Schizophrenics (female)	
ALT (U/L)	26.41 ± 3.76	45.57 ± 4.87	41.97 ± 6.98	48.38 ± 4.98	.010
AST (U/L)	21.92 ± 3.65	40.55 ± 1.34	42.11 ± 4.76	39.45 ± 2.87	.014
ALP (U/L)	83.76 ± 5.87	121.54 ± 4.87	127.53 ± 8.65	131.55 ± 10.65	.018
ALB (mg/dl)	3.61 ± .978	2.71 ± .765	2.79 ± .456	2.64 ± .762	.045
T.Bili (mg/dl)	1.10 ± .056	2.63 ± .987	2.85 ± .564	2.47 ± .675	.034
TP (mg/dl)	6.46 ± 1.65	6.57 ± 1.56	6.58 ± 1.78	6.55 ± 1.33	.087

### Antioxidant Profile

The various antioxidant parameters evaluated demonstrated a correlation between schizophrenics and the development or progression of thyroid disorders. The MDA levels (nmol/ml) varied significantly (*p* = 0.0213) and demonstrated elevated levels (3.71 ± 0.967) in schizophrenics than the healthy group (1.68 ± 0.099) (Table 4). The critical role of ROS and reactive nitrogen species (RNS) in the development of schizophrenia and its progression towards thyroid disorders is evident by the enzymatic and non-enzymatic antioxidants profile including SOD (µg/dl), GSH (µg/dl), CAT (µmol/mol of protein), GPx (mmol/dl) and GR (µmol/ml) respectively. All the above biomarkers showed very significant variations (Table 4). The levels of SOD (0.11 ± 0.0034 Vs. 0.45 ± 0.0056), GSH (4.48 ± 0.965 Vs. 9.06 ± 1.75), CAT (2.30 ± 0.0564 Vs. 3.67 ± 0.0376) and GPx (6.67 ± 0.987 Vs. 8.06 ± 1.87) were reduced in schizophrenics compared to the control. On the other hand, GR levels were higher in the patients relative to control (4.29 ± 0.365 Vs. 1.69 ± 0.002). Pro-oxidant levels including NO, NOS, and oxidative damage products like AOPsP, AGEs, and 8-OHdG showed a significant difference among themselves (*p* = 0.423, 0.017, 0.0110, 0.0287, and 0.0011 respectively). The levels of NO (23.27 ± 3.87 Vs. 56.33 ± 7.45 ng/ml) and NOS (7.87 ± 1.87 Vs. 56.76 ± 2.67 U/L) were decreased, while the levels of AOPPs (1.43 ± 0.0043 Vs. 0.90 ± 0.0067 ng/ml), AGEs (2.77 ± 0.0046 Vs. 2.57 ± 0.0045 ng/ml) and 8-OHdG (2.76 ± 0.0067 Vs. 1.18 ± .0045 ng/ml) were increased in the subjects versus controls.

**TABLE 4:** Antioxidant profiles of schizophrenics versus controls.

Variables	Schizophrenics vs. control (mean ± SD)				*p*-values (**< 0.05)**
	Control	Schizophrenics	Schizophrenics (male)	Schizophrenics (female)	
MDA (nmol/L)	1.68 ± .099	3.71 ± .967	3.77 ± .365	3.66 ± .331	.0213
SOD (µg/dl)	0.45 ± .0056	0.11 ± .0034	0.11 ± .0076	0.11 ± .0056	.0423
GSH (μmol/L)	9.06 ± 1.75	4.48 ± .965	4.13 ± 1.089	4.76 ± 1.78	.0376
CAT (µmol/mol of protein)	3.67 ± .0376	2.30 ± .0564	2.16 ± .076	2.40 ± .067	.0187
GGT (U/L)	44.154.87	57.99 ± 5.87	56.95 ± 6.76	58.79 ± 3.98	.0245
CRP (mg/dl)	1.08 ± .0037	1.45 ± .0022	1.47 ± .0067	1.44 ± .0045	.0354
IL-6 (pg/ml)	3.73 ± 2.76	6.69 ± 3.87	6.61 ± 1.87	6.76 ± .96	.0010
IL-2 (pg/ml)	2.65 ± 1.56	5.34 ± 1.77	6.65 ± 1.87	4.03 ± 1.02	.0310
TNF-α (pg/ml)	30.42 ± 3.87	31.60 ± 5.76	31.78±3.87	31.47 ± 3.08	.0332
AOPPs (ng/ml)	0.90 ± .0067	1.43 ± .0043	1.35 ± .0065	1.49 ± .090	.0110
AGEs (ng/ml)	2.57 ± .0045	2.77 ± .0046	2.77 ± .0031	2.77 ± .0027	.0287
NO (ng/ml)	23.27 ± 3.87	56.33 ± 7.45	56.58 ± 6.64	56.13 ± 7.87	.0423
GPx (μmol/L)	8.06 ± 1.87	6.67 ± .987	6.61 ± 1.78	6.71 ± 1.33	.0187
GRx (μmol/L)	1.69 ± .0023	4.29 ± .365	3.91 ± .0076	4.58 ± .192	.0214

### Cytokine Profile

Cytokines have pleiotropic functions and serve as potential prognostic and diagnostic variables to establish the severity, stage, and treatment of a given disease. The data shown in (Table 4) depicted that cytokines including IL-2, IL-6, and TNF-α play a considerable role in the development of schizophrenia. The levels of IL-2 (pg/ml) (5.34 ± 1.77 Vs. 2.65 ± 1.56), IL-6 (pg/ml) (6.69 ± 3.87 Vs. 3.73 ± 2.76) and TNF-α (pg/ml) (31.60 ± 5.76 Vs. 30.42 ± 3.87) were increased in schizophrenics than the control subjects and varied significantly (*p* < 0.05) among each other.

### Thyroid Hormone Profile

The thyroid function tests and related antibodies exhibited a diverse representation among schizophrenics and control and displayed a statistical significance (*p* < 0.05). Higher levels of freeT4 (21.30 ± 2.87 Vs. 10.66 ± 1.87 pmol/L) and TSH (5.09 ± 1.98 Vs. 2.00 ± 0.0045 mIU/L) and lower levels of freeT3 (3.85 ± 0.99 Vs. 4.58 ± 1.09 pg/ml) were observed in schizophrenics compared to controls (Table 5). Elevated levels of thyroid antibodies also revealed the development of autoimmune thyroid disorders (AITDs) in schizophrenia subjects. The levels of TPO-Ab (IU/ml), TG-Ab (pmol/L), and TSHr-Ab (IU/L) were observed to be higher in the patients’ group as compared to control subjects (9.84 ± 2.56 Vs. 5.81 ± 1.98, 55.50 ± 2.98 Vs. 32.95 ± 2.87 and 2.95 ± 0.0045 Vs. 1.44 ± 0.0023 respectively). The increases in FT4, TSH, and thyroid antibodies in schizophrenics indicate the association of schizophrenia with thyroid disorders.

**TABLE 5:** Thyroid hormone profiles of schizophrenics versus controls.

Variables	Schizophrenics vs. control (mean ± SD)				*p* values (**< 0.05)**
	Control	Schizophrenics	Schizophrenics (male)	Schizophrenics (female)	
FT4 (pmol/L)	10.66 ± 1.87	21.30 ± 2.87	21.36 ± 3.98	21.25 ± 4.65	.0457
FT3 (μg/dl)	4.58 ± 1.09	3.85 ± .99	3.88±.86	3.82 ± .778	.0344
TSH (IU/L)	2.00 ± .0045	5.09 ± 1.98	4.89 ± .997	5.24 ± 1.08	.0214
TgAb (IU/L)	32.95 ± 2.87	55.50 ± 2.98	55.48 ± 5.76	55.51 ± 5.64	.0409
TPOAb (IU/L)	5.81 ± 1.98	9.84 ± 2.56	10.20 ± 3.87	9.55 ± 1.56	.0345
TSHRAb (IU/L)	1.44 ± .0023	2.95 ± .0045	2.94 ± .0034	2.97 ± .0033	.0351

### Vitamins and Trace Elements Profile

The levels of water-soluble vitamins tested in schizophrenia showed significant decrease in their levels compared to the control **(** Table 6 **)**. The decreased levels of Vit B6 (23.98 ± 3.65 nmol/L), Vit B9 (1.97 ± 0.034 nmol/L), Vit B12 (89.98 ± 10.25 pmol/L) and Vit C (0.36 ± 0.0035 nmol/L) were recorded in schizophrenics susceptible to AITDs in comparison to healthy controls (Vit B6 87.67 ± 7.24, Vit B9 2.78 ± 0.092, Vit B12 234.65 ± 11.87 and Vit C 0.54 ± 0.0034 respectively). All the above decreases were statistically significant compared to the control [Vit B6 (*p* = 0.0190), Vit B9 (*p* = 0.0267), Vit B12 (*p* = 0.0023) and Vit C (*p* = 0.0315)]. The levels of fat-soluble vitamins were also reduced during disease severity. The levels of Vit A (4.33 ± 1.62 Vs. 6.01 ± 1.76 nmol/L), Vit E (0.25 ± 0.0035 Vs. 0.28 ± 0.0045 nmol/L) and Vit D (9.57 ± 1.89 Vs. 15.82 ± 1.98 pmol/L) were significantly lower in schizophrenics relative to control (*p* < 0.05) **(** Table 6 **)**. Significantly, reduced levels of trace elements (Se 5.5 ± 0.78 Vs. 4.21 ± 0.02), Zn 0.27 ± 0.0027 Vs. 0.16 ± 0.0023 mg/kg, Cu 0.95 ± 0.0067 Vs. 1.51 ± 0.0026 mg/kg, Fe 1.36 ± 0.0034 Vs. 1.23 ± 0.0093 μmol/L and ferritin 1.83 ± 0.0092 Vs. 1.12 ± 0.0033 μg/L) were recorded in schizophrenics **(** Table 7). As these trace elements play an important role in the proper functioning of anti-oxidative enzymes but their lower levels suggested improper activity of these enzymes which was most likely resulting in oxidative stress-mediated damage.

**TABLE 6:** Vitamin profiles of schizophrenics versus controls.

Variables	Schizophrenics vs. control (mean ± SD)				*p*-values (**< 0.05)**
	Control	Schizophrenics	Schizophrenics (male)	Schizophrenics (female)	
Vitamin A(nmol/L)	6.01 ± 1.76	4.33 ± 1.62	4.42 ± 1.56	4.25 ± .921	.0417
Vitamin C(nmol/L)	0.54 ± .0034	0.36 ± .0035	0.37 ± .0016	0.36 ± .0035	.0315
Vitamin E(nmol/L)	0.28 ± .0045	0.25 ± .0035	0.23 ± .0061	0.26 ± .0026	.0431
Vitamin D(pmol/L)	15.82 ± 1.98	9.57 ± 1.89	9.55 ± 2.87	9.59 ± 1.92	.0271
Vitamin-B6 (nmol/L)	87.67 ± 7.24	23.98 ± 3.65	21.98 ± 2.76	25.98 ± 1.92	.0190
Vitamin-B9 (nmol/L)	2.78 ± .092	1.97 ± .034	1.78 ± .065	2.16± ± .0924	.0267
Vitamin-B12 (pmol/L)	234.65 ± 11.87	89.98 ± 10.25	79.87 ± 11.87	100.09 ± 6.63	.0023

**TABLE 7:** Trace elements profile of schizophrenics versus controls.

Variables	Schizophrenics vs. control (mean ± SD)				P-values (**< 0.05)**
	Control	Schizophrenics	Schizophrenics (male)	Schizophrenics (female)	
Zn (mg/kg)	0.16 ± 0.0023	0.27 ± 0.0027	0.27 ± 0.0014	0.27 ± 0.0025	0.006
Cu (mg/kg)	1.51 ± 0.0026	0.95 ± 0.0067	0.91 ± 0.0053	0.98 ± 0.0063	0.0067
Fe (μmol/L)	1.23 ± 0.0093	1.36 ± 0.0034	1.34 ± 0.0039	1.37 ± 0.0036	0.0454
Ferritin (μg/L)	1.12 ± 0.0033	1.83 ± 0.0092	1.82 ± 0.0023	1.84 ± 0.0024	0.0645
Selenium (ppm)	0.0293 ± 0.000956	0.0075 ± 0.00043	0.0056 ± 0.00073	0.0094 ± 0.00051	0.0319

### Aminoacids and Hormonal Profiles

The facts regarding different amino acids displayed a clear picture of their eminent involvement in the initiation of schizophrenia and its progression to thyroid disorders represented in (Table 8). The levels of sulfur-containing amino acids (homocysteine, cysteine, and methionine) were found to be significantly regulated (Table 8). The homocysteine levels were significantly elevated (*p* = 0.000) in schizophrenics (17.56 ± 2.612 Vs. 6.96 ± 1.987 μmol/L), while cysteine and methionine were significantly decreased (*p* = 0.034 and *p* = 0.014) than the control (1.08 ± 0.089 Vs. 4.87 ± 0.924 μmol/L and 17.87 ± 1.23 Vs. 99.20 ± 5.36 μmol/L). The reason behind this variation may be due to reduced Vit B6, B9 and B12 so that homocysteine can neither be trans-sulfurated nor methylated properly resulting in oxidative stress. The levels of non-essential amino acids (glutamate, arginine, and tyrosine) were markedly reduced in schizophrenics in contrast to the corresponding control (12.70 ± 4.62 Vs. 19.77 ± 3.76 μmol/L, 91.65 ± 3.98 Vs. 95.35 ± 4.76 μmol/L, and 65.90 ± 7.54 Vs. 67.56 ± 3.76 μmol/L) while glycine was increased (267.87 ± 6.63 Vs. 256.73 ± 7.87 μmol/L). The reduction in glutamate levels resulted in oxidative injury while decreased arginine levels depict a fall in NO resulting in neurocognitive decline. The essential amino acids including phenylalanine, leucine, and threonine were higher in schizophrenia patients (64.76 ± 4.98 μmol/L, 155.35 ± 6.64 μmol/L, and 134.35 ± 6.98 μmol/L) relative to the control (63.87 ± 4.87 μmol/L, 145.35 ± 3.87 μmol/L, and 129.76 ± 4.67 μmol/L). The hormonal levels (dopamine, serotonin, and prolactin) were also assessed. Dopamine levels were decreased (3.78 ± 0.92 Vs. 7.98 ± 1.82 pg/ml) while an increasing trend was observed in serotonin and prolactin (PRL) among schizophrenics in comparison to the control (196.98 ± 6.35 Vs. 77.98 ± 7.01 ng/ml and 32.43 ± 1.67 Vs. 15.76 ± 2.65 ng/ml). An increase in serotonin together with a decrease in dopamine is associated with hyperprolactinemia mediated depression (HMD).

**TABLE 8:** Amino acids and hormonal profile of schizophrenics versus controls.

Variables	Schizophrenics vs. Control (Mean ± SD)				*p*-values (**< 0.05)**
	Control	Schizophrenics	Schizophrenics (Male)	Schizophrenics (Female)	
Homocysteine (μmol/L)	6.96 ± 1.987	17.56 ± 2.612	19.56 ± 3.712	15.56 ± 2.87	.000
Glutamate (mmol/L)	19.77 ± 3.76	12.70 ± 4.62	13.02 ± 3.76	12.46 ± 2.78	.0145
Methionine (μmol/L)	99.20 ± 5.36	17.87 ± 1.23	15.54 ± 2.87	20.20 ± 1.97	.0012
Cysteine (μmol/L)	4.87 ± .924	1.08 ± .089	0.99 ± .025	1.17 ± .092	.034
NOS (U/L)	7.87 ± 1.87	56.76 ± 2.67	45.76 ± 3.561	67.76 ± 2.76	.0176
Serotonin (ng/ml)	196.98 ± 6.35	77.98 ± 7.01	69.76 ± 6.26	86.20 ± 7.27	.0017
Dopamine (pg/ml)	7.98 ± 1.82	3.78 ± .92	2.87 ± .729	4.69 ± .623	.0014
Prolactin (ng/ml)	15.76 ± 2.65	32.43 ± 1.67	29.76 ± 2.87	35.10 ± 3.78	.000
8-OHdG (ng/ml)	1.18 ± .0045	2.76 ± .0067	1.98 ± .0032	3.54 ± .0056	.0011
Glycine (μmol/L)	256.73 ± 7.87	267.87 ± 6.63	270.34 ± 9.53	265.40 ± 6.43	.0498
Leucine (μmol/L)	145.35 ± 3.87	155.35 ± 6.64	159.36 ± 3.98	151.34 ± 8.54	.156
Phenylalanine (μmol/L)	63.87 ± 4.87	64.76 ± 4.98	65.36 ± 3.98	63.40 ± 7.54	.287
Tyrosine (μmol/L)	67.56±3.76	65.90 ± 7.54	63.36 ± 2.89	62.54 ± 6.34	.0942
Threonine (μmol/L)	129.76 ± 4.67	134.35 ± 6.98	132.76 ± 4.43	135.94 ± 3.98	.0425
Arginine (μmol/L)	95.35 ± 4.76	91.65 ± 3.98	92.79 ± 6.45	90.51 ± 6.43	.0052

## Discussion

The present study showed that in schizophrenics susceptible to thyroid disorders, deficiency in vitamins B6, B9, and B12 mediates hyperhomocysteinemia resulting in the reduction of antioxidative enzymes leading to oxidative injury. Hyperhomocysteinemia in turn increases asymmetric dimethylarginine (ADMA) levels thereby inhibiting the NOS activity causing the reduction in NO levels, an important mediator in synaptic plasticity and memory. The levels of glutamate and cysteine were lower in schizophrenics, mediating oxidative insult while the decline in arginine levels result in decreased NO levels leading to schizophrenic symptoms. Reduction in Cu and an increase in Zn levels result in lower dopamine and higher serotonin levels leading to hyperprolactinemia mediated depression (HMD). The lower vitamin D levels in schizophrenics may be due to poor nutrition or inadequate sunlight exposure, which in turn increases susceptibility to autoimmune thyroid disorders, as they additionally have raised thyroid antibodies. The increase in thyroid-stimulating hormone (TSH) indicated hypothyroidism while the higher level of H_2_O_2_ resulted in not only hypothyroidism as well as hyperthyroidism (Kivity et al., 2011).

The dietary antioxidants including the vitamins pyridoxine (B6), folic acid (B9), and cobalamin (B12) have gained marked attention as they have a significant role in the prevention of oxidative damage and donation of a methyl group in the production of proteins, lipids, nucleic acids, neurotransmitters, and hormones (Mitchell et al., 2014). It has been proposed that increased levels of vitamins B6, B9_,_ and B12 cause a reduction in homocysteine levels which helps consolidation of working memory. In contrast, their deficiency can result in hyperhomocysteinemia which may induce problems in neurocognitive abilities and behavior (Moustafa et al., 2014). The present study also demonstrated an inverse correlation between homocysteine and vitamins B6 (r = -0.298), B9 (r = -0.523) and B12 (r = -0.498). Thyroid hormones have a positive effect on homocysteine levels by two different mechanisms. Firstly, thyroid hormones can cause a reduction in methionine synthase (MS) and methylenetetrahydrofolate reductase (MTHFR) levels in the liver, involved in the remethylation of homocysteine (Ayav et al., 2005). Secondly, the glomerular filtration rate was most likely reduced by low thyroid hormone levels (Diekman et al., 2001). The effect of these two mechanisms leads to high homocysteine levels (T3 Vs. Homocysteine, r = 0.387, and T4 Vs. homocysteine, r = 0.399).

Hyperhomocysteinemia has a positive effect on ADMA, an inhibitor of NOS, and thus reduces NO levels, a potent vasodilator resulting in microvascular damage in the brain (Keil et al., 2004). The current study also demonstrated an inverse correlation with NOS (Homocysteine Vs. NOS, r = -0.576). This is associated with decreased vascular supply to the brain and thyroid tissue which leads to their atrophy in schizophrenics. Another effect of oxidative stress is lipid peroxidation, in which peroxides (MDA) are produced and combines with NO to produce peroxynitrite in the presence of total plasma peroxidases which further increases lipid peroxidation and causes a reduction in NO levels (Akiibinu et al., 2012), which was similar to the results of the present study (MDA Vs. NO, r = -0.387). NO has an important role in neurotransmission, memory maintenance, cognitive abilities, and synaptic plasticity. Therefore, it usually becomes utilized with a limited amount being available for these vital functions. NO controls the spine growth by mainly involving the postsynaptic regulation of actin cytoskeleton protein through cGMP-PKG cascade which in turn is responsible for actin polymerization in a phosphorylation-dependent manner and is implicated in the synapse formation (Nikonenko et al., 2013). In the case of malnutrition, there is an inadequate intake of L-arginine which results in the deficiency of NO in schizophrenics, as both have a positive correlation (L-arginine Vs. NO, r = 0.299). Malondialdehyde (MDA) reduces membrane stability and also plays a role in DNA damage by forming adducts (Frey et al., 2007). This study reveals a positive correlation between MDA and 8 oxodG, the end product of DNA damage (MDA Vs. 8 oxodG, r = 0.756).

Homocysteine-mediated oxidative stress is considered as one of the significant mechanisms for the toxicity of homocysteine in neuronal cells. In vascular and neuronal cells, auto-oxidation of homocysteine can occur which causes disturbances in redox homeostasis resulting in defective redox signaling mechanisms (Zou and Banerjee, 2005). This effect can be elaborated by increasing ROS production and NO deactivation. In the mitochondria of both astrocytes and thyrocytes, N-acetyl cysteine is converted into cysteine by deacetylation which then reacts with glutamate to form gamma-glutamate-cysteine in the presence of glutamate-cysteine ligase catalytic unit. Gamma-glutamate-cysteine combines with glycine to form glutathione in the presence of glutathione synthetase (Wood et al., 2009). In schizophrenia, amino acids including cysteine and glutamate, and antioxidant enzymes are decreased in astrocytes and thyrocytes simultaneously thus ROS is increased (Erdamar et al., 2008). Glutamate is also a progenitor of NO and a positive correlation was observed between NO and glutamate (NO Vs. glutamate, r = 0.442). Due to an increase in superoxide free radicals, DNA damage causes telomere shortening/erosion which allows the formation of adducts i.e., 8-oxodG and 8-oxoGuo thus result in apoptosis of thyrocytes and astrocytes (Nishioka and Arnold, 2004). This cell death causes ventricular enlargement and hippocampal volume reduction which leads to schizophrenic symptoms and atrophy of the thyroid gland (Nishioka and Arnold, 2004). Hydrogen peroxide is an important factor in thyroid hormogenesis. The increase in H_2_O_2_ enhances the conversion of iodide into iodine, the coupling of monoiodotyrosine (MIT) and diiodotyrosine (DIT), and synthesis of RT3 leads to hyperthyroidism (Chen et al., 2011). On the other hand, it is also suggested that the increase in H_2_O_2_ causes inhibition of iodide uptake and organification which results in hypothyroidism (Moreno et al., 2002). High H_2_O_2_ also induces cell death by activating apoptosis signal-regulating kinase and also favors inflammation (Poljak et al., 2006).

Hyperhomocysteinemia causes cytotoxic effects by reducing antioxidants such as Vit C or E and N-acetylcysteine (Weiss et al., 2003), and the current study demonstrated an inverse correlation of homocysteine with both Vit C (r = -0.498) and E (r = -0.354). Vit C, another antioxidant acts as a cofactor for 5-hydroxytryptophan and dopamine beta-hydroxylase which mediates the conversion of tryptophan to serotonin and dopamine to nor-epinephrine respectively (Cooper, 1961). Dietary lack of Vit C causes a reduction in serotonin levels and also an increase in dopamine levels (Yamamoto and Novotney, 1998) and the current study also showed a positive correlation of Vit C with serotonin (r = 0.343) and an inverse with dopamine (r = -0.459). Vit E acts as an antioxidant to prevent peroxides (MDA) formation involved in lipid peroxidation and thus functions as a membrane stabilizer (Niki et al., 1995). Its levels are markedly reduced in schizophrenic patients, cause lipid peroxidation leading to DNA and cell membrane damage in both thyroid and brain tissues (Petrulea et al., 2012). In the present study, Vit E showed an inverse correlation with both MDA (r = -0.339) and 8-oxodG (r = -0.576). Vit E has a suppressing effect on thyroid activity and also on oxidants thus its supplementation in hyperthyroidism may have a positive role in the reduction of thyroid hormone levels and oxidative damage (Prabakaran et al., 2004). In line with the above, an inverse correlation between Vit E and thyroid hormones (T3, r = -0.221 and T4, r = -0.287) was observed in the present study (Table 9).

**TABLE 9:** Pearsons’ correlation coefficients of prominent variables in the development of thyroid dysfunction in schizophrenics.

Variables	Correlation coefficients	*p* **< (0.05)**
Homocysteine Vs. L-Arginine	−(.676)	0.034
Homocysteine Vs. NOS	−(.576)	0.041
Homocysteine Vs. Vit-E	−(.354)	0.023
Homocysteine Vs. Vit-C	−(.4.98)	0.044
Homocysteine Vs. Vit-B6	−(.298)	0.001
Homocysteine Vs. Vit-B9	−(.523)	0.026
Homocysteine Vs. Vit-B12	−(.498)	0.033
Homocysteine Vs. T3	+(.387)	0.043
Homocysteine Vs. T4	+(.399)	0.020
Homocysteine Vs. MDA	+(.801)	0.019
MDA Vs. 8OHdG	+(.756)	0.017
MDA Vs. Vit-E	−(.339)	0.030
MDA Vs. NO	−(.387)	0.013
Vit-E Vs. T3	−(.221)	0.021
Vit-E Vs. T4	−(.287)	0.034
Vit-E Vs. 8OHdG	−(.576)	0.021
Vit-C Vs. Serotonin	+(.434)	0.041
Vit-C Vs. Dopamine	−(.459)	0.028
L-Arginine Vs. NO	+(.229)	0.042
Cu Vs. Zn	−(.398)	0.011
Cu Vs. Dopamine	+(.427)	0.018
Se Vs. GPx	+(.598)	0.025
Iron Vs. Catalase	+(.644)	0.025
Prolactin Vs. Dopamine	−(.867)	0.002
Prolactin Vs. Serotonin	+(.745)	0.010
Prolactin Vs. IL-6	−(.246)	0.023
Prolactin Vs. IL-2	+(.301)	0.017
Prolactin Vs. TNF-α	+(.465)	0.039
Glutamate Vs. NO	+(.442)	0.045
Zn Vs. NOS	+(.421)	0.018
Zn Vs. SOD	+(.334)	0.041
Vit-D Vs. TPO-Ab	−(.791)	0.006
Vit-D Vs. TSH	−(.673)	0.000
Vit-D Vs. TSHr-Ab	−(.591)	0.028
Vit-D Vs. Tg-Ab	−(.437)	0.019
Vit-D Vs. ALP	−(.391)	0.022
Vit-D Vs. ALT	−(.891)	0.010
Vit-D Vs. AST	−(.403)	0.019
Vit-D Vs. IL-6	−(.721)	0.003
Vit-D Vs. IL-2	−(.614)	0.011
Vit-D Vs. TNF-α	−(.811)	.0190

Schizophrenia and elevated heavy metals have a strong association **(** Wolf et al., 2006; Vural et al., 2010; Liu et al., 2015). The metallothionein, a metal removing protein is required for heavy metals removal, but malfunctioning transcription processes of this protein are reported in schizophrenics (Wolf et al., 2006). The other common factor in schizophrenia is copper (Cu) toxicity which mediates elevated catecholamine oxidation; the resultant end products are harmful hallucinogens (Liu et al., 2015). In contrast, a recent finding revealed that Cu is decreased in schizophrenics (Vural et al., 2010). It is essential for the proper functioning of superoxide dismutase (SOD), dopamine beta-hydroxylase, and tyrosine hydroxylase, and therefore diminished levels of Cu cause oxidative stress and abnormal dopamine nor-epinephrine interaction (Pataracchia, 2008) and we observed a positive correlation for Cu with dopamine (r = 0.427). It has been also proposed that low thyroid function permits heavy metal retention as there is reduced hepatic synthesis of metallothionein (Gupta and Kar, 1983). Heavy metals hamper T4 to T3 conversion by restraining peripheral enzymes (van Vliet et al., 2007). Zinc (Zn) deficiency is an antagonist to Cu (Yanik et al., 2004), the present study also revealed an inverse correlation between these two metals (Cu Vs. Zn, r = -0.398). Zinc is essential for other important functions such as serotonin synthesis, proper functioning of metallothionein, the formation of Cu, Zn, SOD, and activation of NOS. It is important in the prevention of oxidative damage, reduction of lipid peroxidation in neurons, and thus plays a major role in maintaining the blood-brain barrier (Malik, A et al., 2016). The present study suggested that the decreased Cu levels mediate the lowering of dopamine levels while increased Zn levels cause elevated serotonin levels, resulting in hyperprolactinemia leading to hyperprolactinemia mediated depression (HMD), as reported in a recent study (Wysokiński and Kłoszewska, 2014). An inverse correlation between PRL and dopamine (r = -0.867) while a positive correlation between PRL and serotonin (r = 0.745) was observed in the current study. The levels of phenylalanine were increased but that of tyrosine was lowered in schizophrenics. Phenylalanine is converted into tyrosine in the presence of phenylalanine hydroxylase and it ultimately produces dopamine. But in schizophrenia, the activity of this enzyme is affected by cytokines (interferon-γ) and ROS which decreases cofactor 6 R-L-erythroid-5,6,7,8-tetrahydrobiopterin (BH4) required for its proper functioning (Liao and Ya-Mei, 2014). The increase in TSH response to TRH has been recognized, having a direct effect on the thyroid gland resulting in hyperthyroidism (Duval et al., 2010). Contrary to this effect, it is reported that there is a reduction in TSH response to TRH in depression which leads to hypothyroidism (Asare et al., 2014). Selenium (Se) and iron (Fe) deficit are also common in schizophrenics as they act as cofactors for glutathione peroxidase (GPx) and catalase (CAT) respectively thus their deficiency results in oxidative damage (Pataracchia, 2008). The current study demonstrated a positive correlation between Se and GPx (r = 0.598) and also between Fe and CAT (r = 0.664) **(** Table 9).

It has been revealed that individuals with Vit D deficiency are two times more prone to develop schizophrenia, and its deficiency leads to a seasonal affective disorder (SAD), a state of depression that may arise from decreased sunlight exposure and poor nutrition (Bischoff-Ferrari et al., 2006). The levels of antibody titer represent that thyroid peroxidase-antibody (TPOAb), thyroid-stimulating hormone receptor-antibody (TSHrAb), and thyroglobulin-antibody (TgAb) were raised in the patients suffering from schizophrenia along with thyroid dysfunction. A strong inverse relationship between Vit D and the presence of anti-thyroid antibodies in patients deficient in Vit D was revealed (Virupaksha et al., 2014). The present study also represents inverse correlation between Vit D and thyroid antibodies namely TPO-Ab (r = -0.791), TSHr-Ab (r = -0.591) and TG-Ab (r = -0.338*). In the current study, Vit D showed inverse correlation with inflammatory cytokines namely, IL-2 (r = -0.614), IL-6 (r = -0.721) TNF-α (r = -0.811). The enhanced inflammatory response mediates up-regulation of autoimmune response resulting in autoimmune thyroid disorders (AITDs) including Hashimoto’s thyroiditis and Graves’ disease. This decrease in Vit D may be due to deranged liver functions as observed in this study by raised hepatic enzymes including ALT, ALP, and AST, contrary to the findings of Virupaksha et al., 2014 when a high inverse correlation was observed between Vit D and the following hepatic enzymes namely, ALT (r = -0.891), AST (r = -0.403) and ALP (r = -0.391). The deficient 1, 25(OH)_2_D_3_ levels in schizophrenics results in an increase in homocysteine levels by blocking the activity of cystathionine β-synthase (CBS) and also inhibit NOS dependent NO production leading to cognitive decline (Malik, A. A et al., 2016), similar correlations of Vit D with homocysteine (r = 0.502) and NOS (r = 0.411).

**FIGURE 1:**
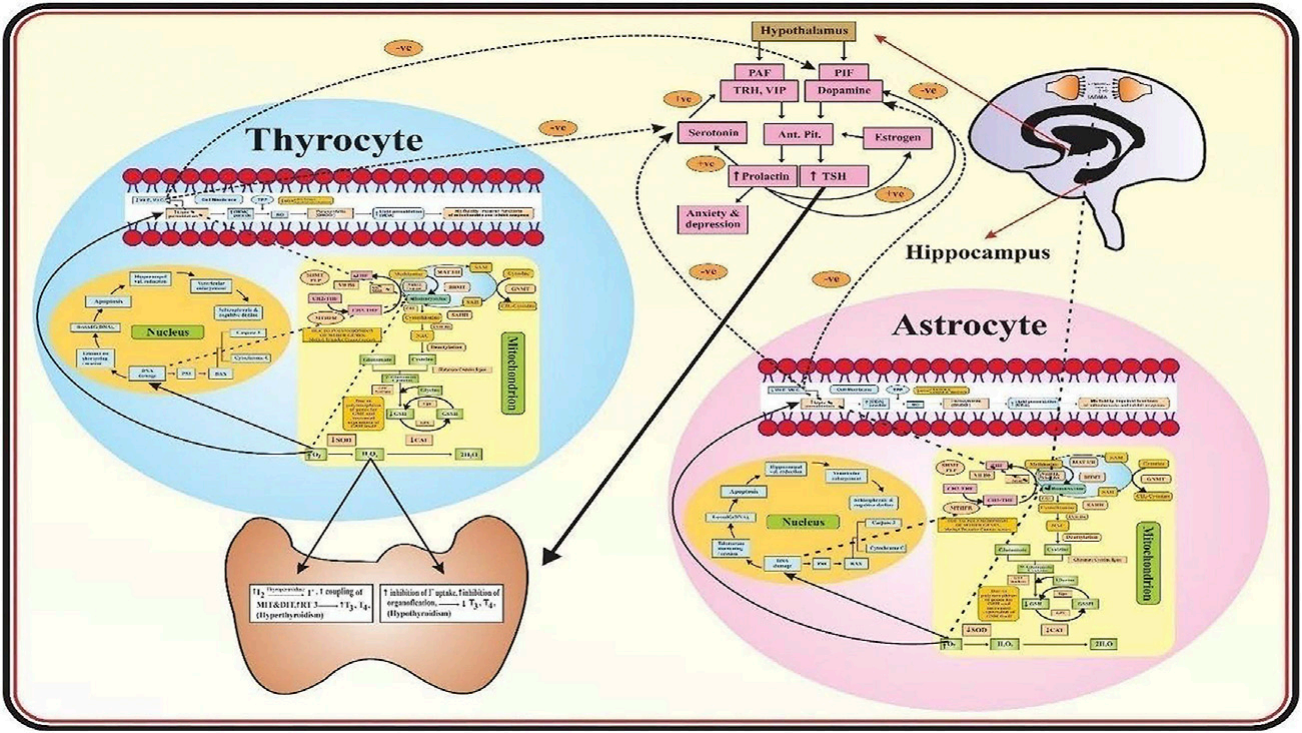
Mechanism of Thyroid Dysfunction in Schizophrenics. The role of oxidative stress and the biomarkers associated with thyroid dysfunction in schizophrenics.

Figure 1 describes that in the mitochondria of astrocytes and thyrocytes, methionine is converted into S-adenosylmethionine (SAM) in the presence of MATI/II which is then converted into S-adenosylhomocysteine (SAH) by glycine N-methyltransferase (GNMT) so that methylation of neurotransmitters occurs. SAH is transformed into homocysteine, mediated by S-adenosylhomocysteine hydrolase (SAHH). Homocysteine has two routes, one is to remethylate into methionine in the presence of methionine synthase (MS), vitamin B9 and vitamin B12 act as a cofactor for it. The methyl group is provided by methyltetrahydrofolate (CH3-THF), formed from tetrahydrofolate (THF) in the presence of serine hydroxymethyltransferase (SHMT), methyltetrahydrofolate reductase (MTHFR), and vitamin B6. The other route is that homocysteine is converted into cystathionine-by-cystathionine synthase beta (CSβ) and then into N-acetylcysteine (NAC) which requires vitamin B6 as a cofactor. NAC is then converted into cysteine by deacetylation which then combines with glutamate and glycine to form glutathione (GSH). In schizophrenia, glutathione is reduced due to polymorphism of its gene, and deficiency of vitamin B6, B9, and B12 may be due to reduced dietary intake, malabsorption, and genetic dysfunction. Thus, it leads to hyperhomocysteinemia which causes oxidative stress, vascular damage, DNA damage, mitochondrial dysfunction, and apoptosis of both astrocytes and thyrocytes. Furthermore, in schizophrenics, antioxidant enzymes such as SOD, GPx, and CAT are also reduced. Thus, there is a dual cause of an increase in oxidative stress. This causes DNA damage and apoptosis leading to hippocampal volume reduction and ventricular enlargement in the brain and thyroid atrophy as well. Lipid peroxidation produces peroxide (MDA) which forms peroxynitrite when combined with nitric oxide (NO) in the brain coming from blood vessels, produced by L-arginine and oxygen in the presence of nitric oxide synthase (NOS). NO has an important role in vasodilation, memory maintenance, neurotransmission, and cognition. NO synthesis is affected in schizophrenia by hyperhomocysteinemia by increasing asymmetrical dimethyl aspartate (ADMA), an inhibitor of NOS, and also due to L-arginine dietary lack. Thus, microvasculature is destroyed resulting in decreased blood supply to the brain and thyroid tissues proceeding to apoptosis. The peroxides (MDA) cause DNA damage which ultimately leads to astrocytes and thyrocytes apoptosis. In thyrocytes, hydrogen peroxide (H_2_O_2_) is used in the synthesis of thyroid hormone as it acts on thyroperoxidase (TPO) mediated reactions. Thus, the increase in H_2_O_2_- causes hyperthyroidism (Song et al., 2007). The rise in H_2_O_2_ causes inhibition of iodide uptake and organification resulting in hypothyroidism (Song et al., 2007; Szanto et al., 2019). In the hypothalamus, prolactin (PRL) activating factors (TRH and VIP) and PRL inhibiting factor (dopamine) are released which act on the anterior pituitary to produce PRL and TSH. In schizophrenics, hyperprolactinemia occurs as PRL increases estrogen which induces more production of PRL. PRL also enhances serotonin synthesis which increases PRL activating factors and reduces dopamine, thus more hyperprolactinemia occurs leading to anxiety and depression. TSH is also increased and acts on the thyroid resulting in hyperthyroidism. Hyperhomocysteinemia also reduces the levels of vitamin C and E, the dietary antioxidants in schizophrenia. The reduction in vitamin C causes a decrease in serotonin and dopamine as it acts on enzymes that mediate their synthesis and processing. Vitamin E reduces lipid peroxidation thus has a significant role in membrane stabilization. Hence, the reduction in vitamin E may substantially elevate the lipid peroxidation levels resulting in mitochondrial and cell membrane damage and apoptosis of astrocytes and thyrocytes in schizophrenics.

## Conclusion

In the present study, schizophrenics demonstrated altered liver function, increased stress markers, and decreased dietary antioxidants. The reduced levels of primary and secondary antioxidants may subsequently result in hyperhomocysteinemia and increased ROS leading to DNA and mitochondrial damage. Moreover, we found that in schizophrenics susceptible to thyroid disorders, deficiency in vitamins B6, B9, and B12 mediates hyperhomocysteinemia resulting in the reduction of antioxidative enzymes leading to oxidative injury. Hyperhomocysteinemia in turn increases ADMA levels thereby inhibiting the NOS activity causing a decrease in NO levels, an important mediator in synaptic plasticity and memory. The levels of glutamate and cysteine were lower in schizophrenics, mediating oxidative insult while the decline in arginine levels result in decreased NO levels leading to schizophrenic symptoms. Besides, the reduction in Cu and an increase in Zn levels result in lower dopamine and higher serotonin levels leading to HMD. Also, the lower vitamin D levels in schizophrenics increase their susceptibility to autoimmune thyroid disorders. In conclusion, homocysteine and/or PRL levels may serve as candidate prognostic markers for schizophrenia. Besides, both neurological symptoms and the susceptibility to thyroid disorders may be prevented in the initial stages of this debilitating disorder by appropriate dietary supplementation of antioxidants which can rectify a reduction in primary and secondary antioxidants, and disturbed prolactin-serotonin-dopamine interactions in schizophrenics.

## Data Availability

The raw data supporting the conclusions of this article will be made available by the authors, without undue reservation.
